# Freshwater spreading far offshore the Japanese coast

**DOI:** 10.1038/s41598-024-63275-6

**Published:** 2024-06-24

**Authors:** Taku Wagawa, Yosuke Igeta, Kei Sakamoto, Marika Takeuchi, Shinobu Okuyama, Shoko Abe, Itsuka Yabe

**Affiliations:** 1 Fisheries Resources Institute, Japan Fisheries Research and Education Agency, Niigata, 951-8121 Japan; 2 Japan Meteorological Agency, Tokyo, 105-8431 Japan; 3 National Oceanography Centre, Southampton, SO14 3ZH UK; 4 Akita Prefectural Institute of Fisheries, Oga, 010-0531 Japan; 5 Atmosphere and Ocean Research Institute, The University of Tokyo, Kashiwa, 277-8564 Japan; 6 Department of Ocean Sciences, Tokyo University of Marine Science and Technology, Tokyo, 108-8477 Japan

**Keywords:** Coastal water, Sea of Japan, Salinity, Water discharge, Chlorophyll-*a*, Biological production, Ocean sciences, Physical oceanography

## Abstract

River discharge to the ocean influences the transport of salts and nutrients and is a source of variability in water mass distribution and the elemental cycle. Recently, using an underwater glider, we detected thick, low-salinity water offshore for the first time, probably derived from coastal waters, in the central-eastern Sea of Japan, whose primary productivity is comparable to that of the western North Pacific. Thereafter, we aimed to investigate the offshore advection and diffusion of coastal water and its variability and assess their impact. We examined the effects of river water discharge on the flow field and biological production. Numerical experiments demonstrated that low-salinity water observed by the glider in spring was discharged from the Japanese coast to offshore regions. The water is discharged offshore because of its interaction with mesoscale eddies. A relationship between the modeled low-salinity water transport to the offshore region and the observed chlorophyll-*a* in the offshore region was also observed, indicating the influence of river water on offshore biological production. This study contributes to understanding coastal-offshore water exchange, ocean circulation, elemental cycles, and biological production, which are frontiers in the Sea of Japan and throughout the world.

## Introduction

**Figure 1:**
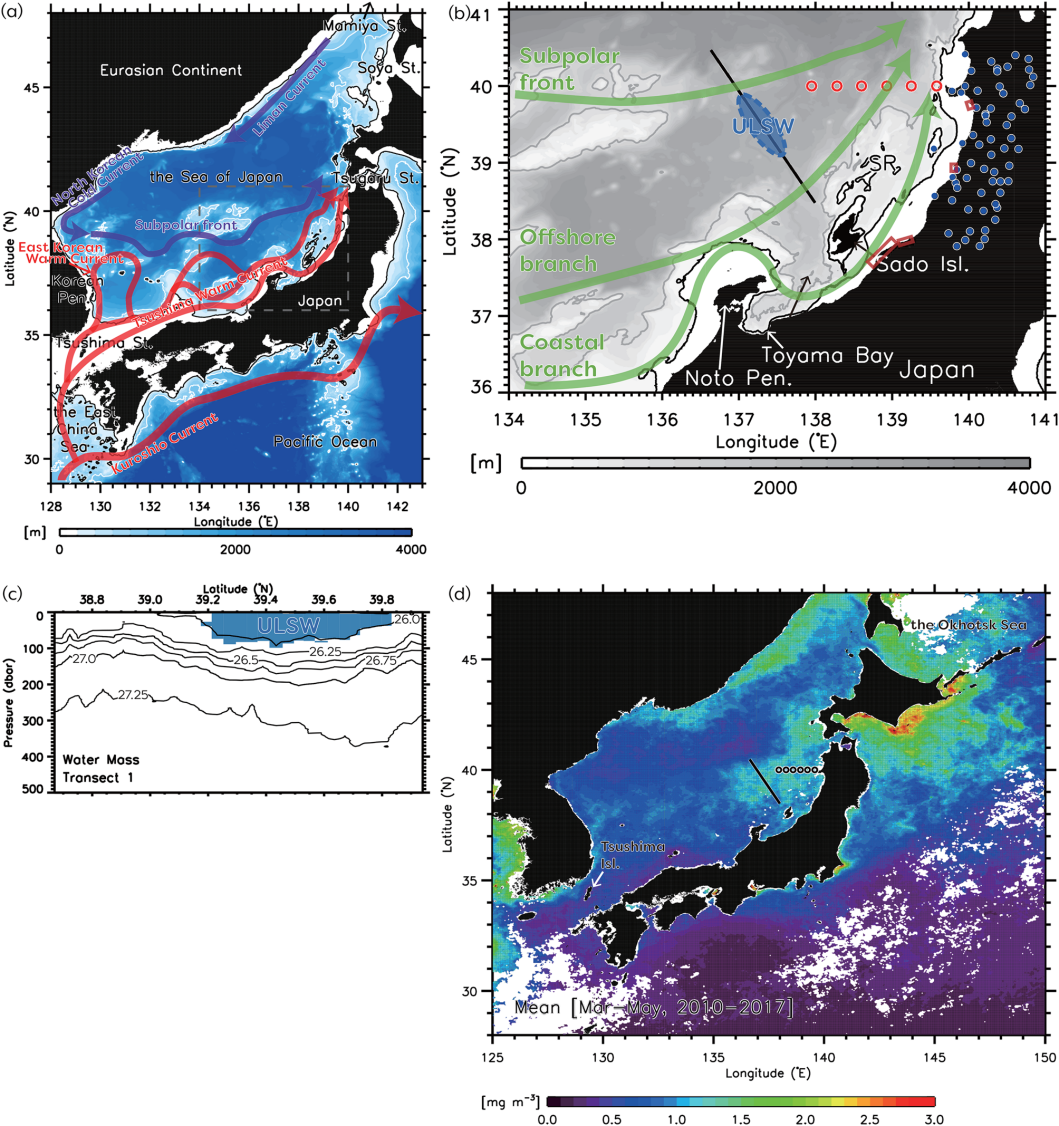
(**a**) Bottom topography and schematic ocean currents around Japan based on the study by Yabe et al.^13^. (**b**) Schematic diagram depicting the path of jets around the study region (green arrows; the coastal/offshore branches of the Tsushima Warm Current and the subpolar front) based on Naganuma^25^ and Wagawa et al.^26^ and the upper-layer low-salinity water (ULSW) (blue ellipse) observed using an underwater glider in Wagawa et al.^26,27^ (black line). Contours of 200 m and 1000 m depths are denoted respectively by black and gray contours. The red rectangles denote the mouths of the major rivers, in order from north to south: the Omono, Mogami, Agano, and Shinano Rivers. Red open circles and blue circles represent the locations of CTD and AMeDAS stations, respectively. The abbreviation for the topography name is SR, Sado Ridge. (**c**) Vertical cross-section of the upper-layer low-salinity water (ULSW, blue) from the glider survey in Wagawa et al.^26^ with salinity and potential density definitions of Wagawa et al.^27^. Contours represent the potential density (kg m^−3^). (**d**) Springtime (March–May) mean sea-surface chlorophyll-*a* (mg m^−3^) averaged during 2010–2017 from satellite color measurement. Black lines and circles represent the glider track and CTD stations, as in (**b**). IDL 9.0 (https://www.nv5geospatialsoftware.com/Products/IDL) is used in generating the bathymetry based on the 1-km JTOPO30 data set provided by the Marine Information Research Center of the Japan Hydrographic Association (www.mirc.jha.or.jp/products/JTOPO30v2/).

The Sea of Japan (SJ), a marginal sea (< 3700 m depth) of the northwestern Pacific Ocean, is bounded by an Asian landmass and the Japanese Islands. It is called a ‘‘miniature ocean’’ because it has a western boundary current that flows along the Korean coast^1–4^ (Fig. 1a). Moreover, cold bottom water was generated in northern SJ during winter^5,6^. The dynamic characteristics of the SJ resemble those of the global ocean^7^. The SJ communicates with the Pacific Ocean through the East China Sea, the Okhotsk Sea (Fig. 1d), and the Tsugaru Strait. Warm, saline Kuroshio water enters the SJ via the Tsushima Strait as two jets along either side of Tsushima Island (Fig. 1d): the East Korea Warm Current and the Tsushima Warm Current via the western and eastern channels, respectively^8–10^. The East Korea Warm Current (a western boundary current along the coast of the Korean Peninsula) separates from the Korean coast and subsequently forms a subpolar front extending roughly along 40° N to the Tsugaru Strait^4,11–13^ (Fig. 1a). The Tsushima Warm Current, which is fundamentally driven by sea level differences between the Tsushima and Tsugaru Straits^14,15^, flows northeastward along the coast of Japan^16^.

Low-salinity, nutrient-rich river discharge alters ocean circulation, water masses, and elemental cycles^17,18^. In eastern Japan, on the SJ side, four rivers flow into the coastal seas, which rank among the top five in Japan in terms of discharge^19^ (Fig. 1b). Moreover, this area receives heavy snowfall, and increased river water from snowmelt, especially in spring, flows out from the land into the ocean, affecting the marine environment in coastal areas^20,21^. River water supplies nutrients and enhances lower-order biological production, such as the primary production of phytoplankton and the secondary production of zooplankton, which support essential fishes in the SJ, such as the Japanese common squid and yellowtail^22–24^.

Via observational studies using an underwater glider, we found that river water from Japanese coastal areas may have been discharged far into the offshore area of the SJ in spring^26^ (Fig. 1c). We suggested that this low-salinity water is not directly affected by Changjiang Diluted Water^8,28, 29^, which flows into the East China Sea at 31.5° N, 122° E and along the Japanese coast, because Changjiang Diluted Water disappears in November–March due to vertical mixing. This result raises the question of the process of coastal water discharge to offshore areas because coastal water is generally trapped on the coast owing to topographic and Coriolis effects^30^, unless it is a large river such as the Changjiang River. Central-eastern SJ is characterized primarily by mesoscale northeastward flows along the subpolar front as a part of the subpolar circulation, the coastal branch of the Tsushima Warm Current and the offshore branch of the Tsushima Warm Current (OBTWC) originating from the Kuroshio (Fig. 1a,b). For coastal waters to spread offshore, they need to spread in a way that crosses these currents; this process is not simple from the conventional understanding and requires a new perspective. Therefore, it is necessary to understand the spreading process that disrupts the dynamic background balance.

Several studies have been conducted on the effects of coastal water on coastal flow fields and ecosystems in the inner waters of the Tsushima Warm Current; however, these studies are usually restricted to areas within 10 km^31,32^. The impact of coastal waters on the offshore ocean, overcoming boundary flow systems, has been an issue for many years because alongshore currents dominate the flow fields in coastal and shelf systems. They also have limited methods for examining them because it is difficult for field measurements and numerical experiments to cover a sizable coast-offshore region with high resolution. Only recently have some studies on coastal-offshore interactions and cross-shore currents been conducted to explore this frontier. For example, Androulidakis et al.^33^ investigated how eddies transport coastal water to offshore areas in the Gulf of Mexico. Malan et al.^34^ investigated the characteristics of the eddy-driven cross-shelf exchange in the East Australian Current system. These studies are necessary because they show that coastal waters can influence ocean circulation and ecosystems in offshore areas approximately 100 km away from the coast. However, case analytical studies examining the processes of ephemeral phenomena have not been extensively conducted. Urakawa et al.^35^ carried out a realistic numerical experiment and suggested that freshwater inputs from rivers affect salinity distribution far from their mouths over the surrounding boundary flows. From a budget-based approach, Malan et al.^34^ showed that cross-shelf currents are prominent in the Eastern Australian Current waters, to some extent, robustly from a climatological perspective rather than an event-like picture using a numerical model and suggested their impact on biological productivity.

It is evident that river water affects coastal areas^36,37^. However, if the water is discharged far offshore, its impact is substantial. It has been suggested that the offshore region of the central-eastern SJ has primary productivity in spring comparable to that of the North Pacific subarctic region, one of the most productive areas in the world^38^ (Fig. 1d). This high productivity may be related to the offshore spread of coastal waters.

Because turbid coastal water prevents sunlight, primary production is only active in the upper layers of coastal waters, and nutrients are not exhausted in the lower layers of the East China Sea^39^. The nutrients in the lower layer can move far offshore until they encounter sufficient light for primary production. This results in high primary production over a wide area. However, the problem is simpler because the offshore discharge is along an eastward current as background flow. This study’s problem is more complex because offshore spreading must cross a strong background current, as mentioned above.

The glider research by Wagawa et al.^26^ captured low-salinity water in the offshore region during spring 2016 for the first time because it was not affected by severe weather conditions in the SJ during spring, which render ship observations difficult. The next stage of this research is to understand river water’s offshore advection and diffusion process, explore its variability, and evaluate its impact. However, the spatiotemporal coverage and resolution of field observations alone are insufficient for a detailed analysis. Therefore, high-resolution numerical models that accurately reproduce river discharges and oceanic fluctuations should be used.

We aimed to investigate the three-dimensional structure and physical mechanism of river water discharge from coastal areas to offshore areas using numerical experiments and field observations and to evaluate the effects of river water discharge on the flow field and biological production. Numerical experiments can also investigate whether the low-salinity water captured by glider observations in spring 2016 was representative of the generic conditions that persisted only in that year or had occurred in other years as well. This study focused on the interannual variability of discharge and investigated the process utilizing detailed comparisons between years in which the discharge was large and those in which it was not.

## Results

### Freshwater spreads offshore far away from the coast

**Figure 2:**
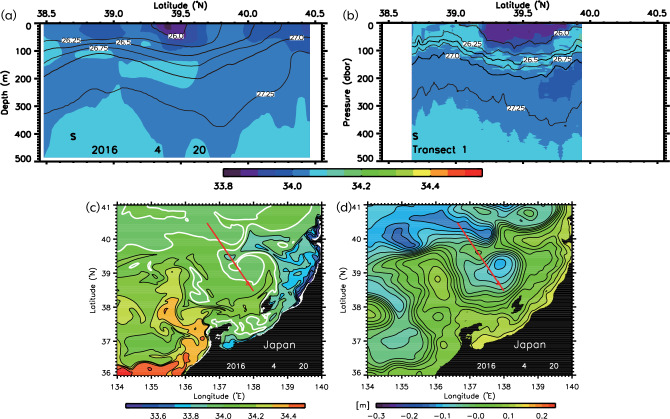
(**a**) Model vertical cross-sections of salinity (color) on 20 April 2016. (**b**) Observed vertical cross-section of salinity (color) during April 20–27, 2016 by the glider survey of Wagawa et al.^23,26^. Contours show potential density (kg m^−3^). Model the upper-layer salinity at a depth of 40 m (thick white contours: salinity of 34.0) (**c**), SLA (**d**). IDL 9.0 (https://www.nv5geospatialsoftware.com/Products/IDL) is used in generating the bathymetry based on the 1-km JTOPO30 data set provided by the Marine Information Research Center of the Japan Hydrographic Association (www.mirc.jha.or.jp/products/JTOPO30v2/).

The vertical cross-section was reproduced using the reanalysis dataset of MOVE/MRI. COM-JPN, generated using the ocean model data assimilation system, validated the upper-layer low-salinity water (ULSW) (Fig. 2a) captured by glider observations in April 2016^26^ (Fig. 2b). Estimating the area of water with salinity less than 34.0 at depths shallower than 100 m, the results of the numerical experiments (Fig. 2a) are 97.8% of the observed results (Fig. 2b), and it can be judged that the distribution of low salinity water is acceptably reproduced. The horizontal distributions of the upper-layer salinity (ULS) at a depth of 40 m and the sea level anomaly (SLA) reproduced by the model data suggest that the ULSW originated from coastal water that flowed out from Japanese coastal areas to the sea and was then advected and diffused as far as 200 km away (Fig. 2c,d). It appears to have been transported from southeast to northwest (Fig. 2c) along the southern side of the anticyclonic eddy centered around 40° N, 139° E (Fig. 2d).

**Figure 3:**
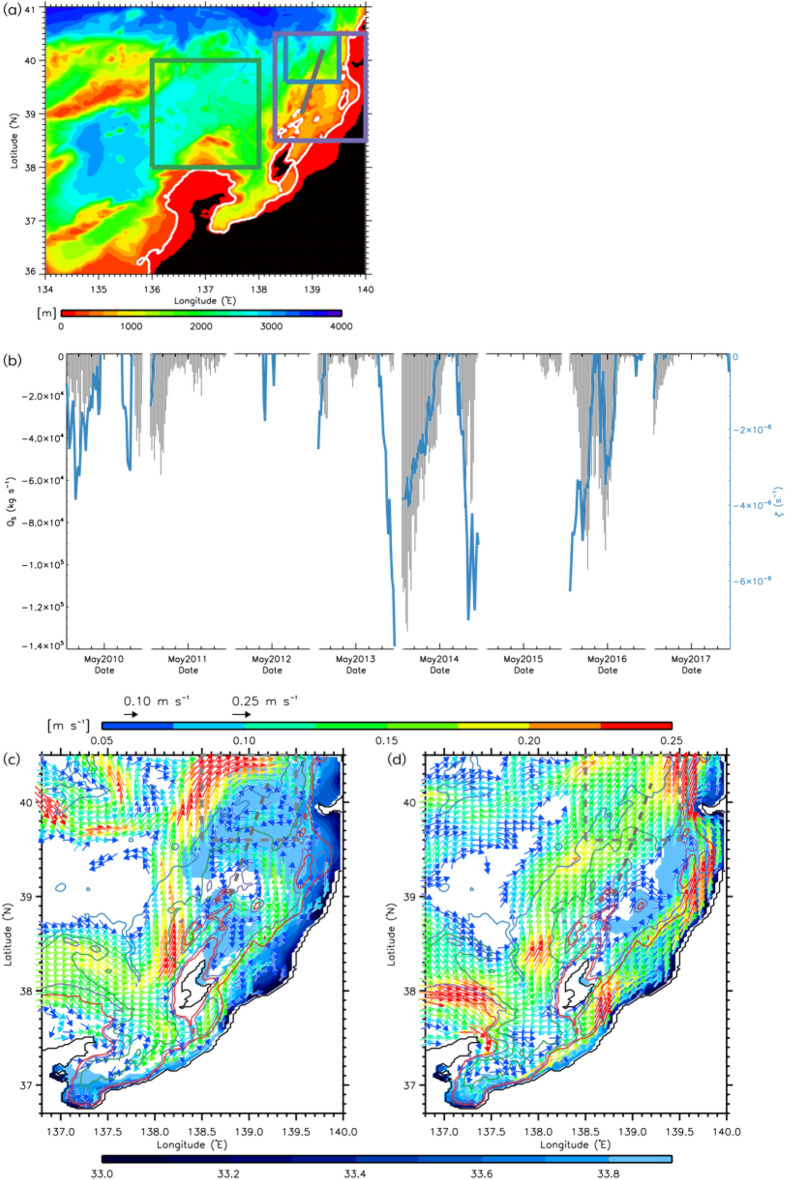
(**a**) Model bottom topography around the study region. The green and blue rectangles indicate the region where the sea-surface chlorophyll-*a* and the longitude of the OBTWC path are estimated in Fig. 4b. Contours of 200 m depth are denoted by white contours. (**b**) Model time series of northwestward salt transport with reference salinity of 34.0 (*Q*_*S*_; kg s^−1^) across the gray line of (**a**) (black bars) and relative vorticity averaged over the blue rectangle of (**a**) (blue lines) during April–May. Model composite average of the sea-surface velocity vectors greater than 0.05 m s^−1^ (arrows) and sea-surface salinity lower than 33.9 (color) when *Q*_*S*_ ≤  − 7.0 × 104 kg s^−1^ (c) and − 0.9 × 104 kg s^−1^ ≤ *Q*_*S*_ ≤  − 0.7 × 104 kg s^−1^ (**d**). Contours of 200 m, 400 m, 1000 m, and 2000 m depths are presented respectively as red, purple, green, and blue contours. The dashed line and rectangle are the same as the gray line and blue rectangle of (**a**). IDL 9.0 (https://www.nv5geospatialsoftware.com/Products/IDL) is used in generating the bathymetry based on the 1-km JTOPO30 data set provided by the Marine Information Research Center of the Japan Hydrographic Association (www.mirc.jha.or.jp/products/JTOPO30v2/).

**Figure 4:**
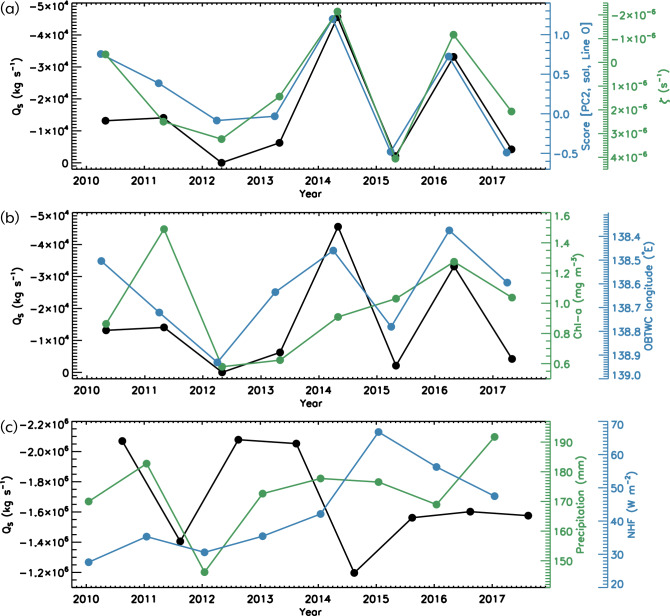
(**a**) Time series of interannual variations of the northwestward salt transport with reference salinity of 34.0 (*Q*_*S*_) averaged during April–May (black), observed standardized the second principal component (PC2) score of salinity along the ship-based CTD line in April (red circles in Fig. 1b; blue), and relative vorticity averaged over the region represented by the blue rectangle in Fig. 3a during April–May (green). (**b**) Time series of interannual variations of the same salt transport as (**a**) (*Q*_*S*_; black), observed sea-surface chlorophyll-*a* averaged over the green rectangle of Fig. 3a during April–May (green) and observed longitude of the offshore branch of the Tsushima Warm Current (OBTWC) path averaged over the purple rectangle of Fig. 3a during March–May (blue). (**c**) Time series of interannual variations of model northeastward salt transport across the Tsushima Strait with reference salinity of 34.0 (*Q*_*S*_) averaged during July–September (black), observed precipitation (including snowfall) over Akita and Yamagata Prefectures (blue circles in Fig. 1b), Japan averaged during December–February (green), and model sea-surface net surface heat flux (positive values represent upward flux) averaged over 36° N–41° N, 134° E–140° E during January–March (blue).

To quantitatively indicate the offshore spread of coastal freshwater, we defined salt transport (*Q*_*S*_) as

$${Q}_{S}=\iint \rho \left(S-{S}_{0}\right){v}_{NW}dxdz,$$

where the positive *x* and *y* axes run along the cross-sectional (thick gray line in Fig. 3a) directions, respectively, *ρ* is the density, *S* is the salinity, *S*_0_ is the referenced salinity (= 34.0), and *v*_*NW*_ is the velocity in the *y* direction (northwestward) during April–May. We integrated in the vertical section of thick grey line in Fig. 3a and only at points where *v*_*NW*_ was northwestward, *S* was less than 34.0, and *Q*_*S*_ was negative. The large amplitude of negative *Q*_*S*_ is indicative of low-salt transport. We hereafter refer to the large (small) amplitude of negative *Q*_*S*_ as “large (small) low-salt transport”. Comparing the spread in 2016 with other years, it is clear that 2016 and 2014 had a substantially higher spread (Fig. 3b). To investigate the effect of anticyclonic eddies on this spread, we compared the spatially averaged over the regions where anticyclonic eddies frequently formed (the blue rectangle in Fig. 3a and gray dashed rectangles in Fig. 3c,d); relative vorticity with salt transport and found that the relative vorticity was more (less) negative when low-salt transport was large (small) in 2014 and 2016 (2012 and 2015) (Fig. 3b). The interannual variations of these *Q*_*S*_ and the spatially averaged relative vorticity averaged over April–May were in good agreement (Fig. 4a). The degrees of freedom were small (*N* = 8) because the model only ran for a limited number of years; however, a significant correlation between *Q*_*S*_ and the relative vorticity (black and green lines) was observed (*r* = 0.89, *P* < 0.01).

The composite sea-surface salinity and velocity vectors were divided into periods of large and small low-salt transport (25 days in each period) to characterize the horizontal distribution of flow and salinity (Fig. 3c,d). A vortex-pair structure consisting of the anticyclonic eddy centered around 40° N, 139° E and the cyclonic eddy to the south of it was clearly extracted when low-salt transport was large. We found that when low-salt transport was large (small), the OBTWC was located further offshore (coastal) and was strong. We examined the relationship between offshore spreading from the model and the path of the OBTWC using satellite observation data. The blue line in Fig. 4b shows the observed longitude of the Tsushima Warm Current path during March–May over the region where the OBTWC frequently flows^13^ (the purple rectangle in Fig. 3a), which can be identified as a continuous flow path using a recently developed flow path determination algorithm^13^. These results are consistent with the scenario (*r* = 0.76, *P* < 0.05), that is, when the offshore spread of low-salinity water is large, the OBTWC flows more to the west.

**Figure 5:**
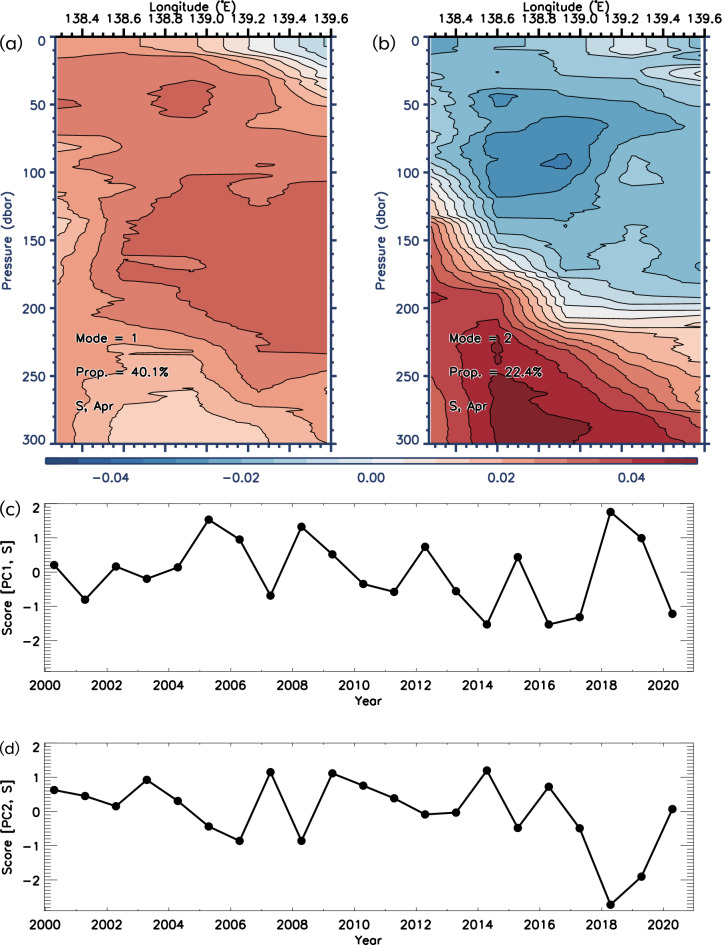
(**a**) Vertical sections of the first (**a**) and second (**b**) principal components (PC1 and PC2) for the ship-based CTD salinity in April (red circles in Fig. 1b) and their standardized scores (**c,d**).

Here, we further analyzed the non-assimilated observed data and pursued consistency with the results of model data. We performed principal component analysis (PCA) of the ship-based CTD salinity vertical section (red circles in Fig. 1b). PCA was performed using the correlation matrix method. In April, the first (second) mode explained 40.1% (22.4%) of the standardized variability in salinity. These components were separated according to the rules of thumb proposed by North et al. (^40^). Figure 5a,c show the vertical sections of the first principal component (PC1) of the interannual variation in salinity in April. Almost all salinities of PC1 (Fig. 5) were in phase with a weaker ULS frontal structure than that of PC2 (Fig. 5b) near the coast centered around 139.2° E.

The second principal component (PC2) seemed to show variability in the offshore spread of coastal water (Fig. 5b,d). Low-salinity water occupies the upper layers of the coastal area, and a strong salinity front forms between it and the relatively high-salinity water in the offshore subsurface regions. A quantitative comparison showed good agreement between the variations of the modeled salt transport and the observed salt section (Fig. 4a; *r* =  − 0.87, *P* < 0.01). It should be noted that these CTD data were not assimilated into the model. Thus, the good agreement between the models and observations is significant.

### Background conditions preventing low-salt transport offshore

Figure 4c shows the interannual variations in preconditions considered unfavorable for the offshore spreading of coastal water. They can potentially block the spread of coastal water. Freshwater discharge from Changjiang River is known to flow into the East China Sea at 31.5° N, 122° E and along the Japanese coast, and for maximum discharge during summer–autumn^8,28, 29^ (Fig. S1). If the inflow defined as northeastward salt transport across the Tsushima Strait with a reference salinity of 34.0 from the model (black line) is weak, it would be difficult for Japanese coastal water to spread offshore due to the adjustment of the geostrophic current with a large horizontal density gradient. Moreover, if the previous winter sea surface mixing derived by atmospherically forced cooling (blue line) were intense, mixing with more saline water in the subsurface layer could favor an increase in salinity of the surface layer, again promoting the geostrophic adjustment. Furthermore, if river water discharge near the Japanese coast as a source of coastal water fluctuation by precipitation (green line) is weak, it might also be difficult for the coastal water to spread. The results qualitatively showed that spreading tended to be weak in years when at least two or more of these adverse conditions coincided (Fig. 4c). In 2012, the inflow of freshwater from the Changjiang River in the last summer and autumn was weak, with low precipitation and snowfall in the preceding winter resulting in unfavorable conditions for the spread. In 2015, the weak inflow of the Changjiang diluted water and the intense surface mixing in the previous winter resulted in poor spreading conditions. In the present study, these three conditions were treated as independent, but perhaps there is a relationship between them. This is a subject for future works.

### Impact of coastal water on primary production

We investigated the impact of coastal water on primary production from observational data of the merged ocean color image of chlorophyll-*a* concentration appeared in a rectangular area when coastal water spreading was suggested to occur frequently (Fig. 4b). There was a good qualitative correspondence between the modeled salt transport and the observed chlorophyll-*a* fluctuations, with maxima in 2016 and 2011 and minima in 2012, although this was not the case for 2014 and 2015. This finding is important because it suggests the primary productivity contribution using observational data. This also shows that coastal waters promote high-nutrient water and accelerate phytoplankton blooms (Fig. 1d).

## Discussion

To investigate the influence of the important factors controlling the light conditions in the offshore area on primary production, the spatially averaged surface photosynthetically available radiation (PAR)^41^ and vertical diffuse attenuation coefficient at the wavelength of 490 nm (*K*_*d*_)^42^ in the same area where the chlorophyll-*a* spatial mean was calculated (green rectangle in Fig. 3a) were analyzed. The variation over time of the high/low surface PAR could induce high/low sea-surface chlorophyll-*a*. *K*_*d*_ is related to light penetration and availability inside the water column and is a good indicator of turbidity. Large/small *K*_*d*_ corresponds to high/low turbidity, which tends to result in low/high sea-surface chlorophyll-*a*^43^. For example, in 2016, when chlorophyll-*a* was high, PAR was relatively high, whereas *K*_*d*_ was minimal (Fig. S2), which did not lead to the conclusion that the light availability enhanced chlorophyll-*a*. In contrast, the relatively large chlorophyll-*a* observed in 2015 and 2017 (Fig. 4b) despite the maxima in low-salt transport, strengths of the anticyclonic eddy and OBTWC (Fig. 4a,b) may be related to the fact that PAR and *K*_*d*_ were maximal (Fig. S2).

Nevertheless, the results also show that the light availability was poor (Fig. S2) in 2011 when chlorophyll-*a* was at its maximum (Fig. 4b). Thus, it was not possible to conclude from the results of this study solely whether the light availability in the offshore area itself affected primary production. We are planning to directly observe this aspect during April–June using a glider with a chlorophyll-*a* sensor to ensure that we are able to resolve some of the unknowns regarding light availability.

To investigate the possible influence of vertical transport of nutrients because of wind-driven mixing in the offshore area on the offshore chlorophyll-*a*, the influence of near inertial waves^44,45^ was investigated using a slab model^46^. As the tides were small and negligible in this area^45,47^, only the effect of wind-driven near inertial waves was considered. There appeared to be no near inertial wave generation because of atmospheric disturbances that could lead to water mass mixing, which could explain the variability in chlorophyll-*a* (Fig. S3).

Upwelling driven by strong currents impinging on steep bathymetry could also be a factor in nutrient supply to the surface. However, as seen in Figs. 1b and 3a, this is unlikely to be the case in the area of the Noto Peninsula and Sado Island, as the currents and bathymetry in this area are not such.

It is generally considered that nutrients may have been isopycnally transported from other regions; however, this has not been substantiated by an earlier study^26^. Nutrient-rich Changjiang diluted water transported from the west is dissipated by surface mixing in winter, and surface high-nutrient waters in the subarctic region flowing to the north could not explain the low-salt, high-chlorophyll-*a* water in the surface layer in the present study, because they are located in much deeper layers when sub-ducted to the south into this area^26^.

Considering the possibility of upwelling because of the divergence of horizontal currents in the offshore area itself, the mean divergence in the same area as the chlorophyll-*a* spatial mean during April–May is of the order of *O*(10^–8^) s^−1^, a sense of upwelling. The maximum spatial mean divergence of interannual variability, not the mean, is of the order of *O*(10^–7^) s^−1^. This value is smaller by an order of magnitude than the value of approximately 0.5 × 10^–5^ s^−1^, which has been shown to be the value of significant divergence in previous studies^48^. Approximately estimated for the present case, 1.0 × 10^–7^ s^−1^, with a mixed layer of 50 m, the upwelling is 5 × 10^–6^ m s^−1^, which is negligible as it only carries water upwards by approximately 0.4 m in a 24 h period.

According to Schiller et al.^49^, the direct wind-drag effect is important for the advection and diffusion of low-salinity water over the continental shelf. Analysis of the wind stress data, which is also used to force the numerical experiments in the present study, shows that there were no significant south-easterly or north-easterly winds on the continental shelf, which implies that the wind did not drag the water over the continental shelf and transport it offshore (not shown).

It is known that upwelling is driven by submesoscale internal waves and frontgenesis^50^. To investigate their effects, the submesoscale divergence and the lateral strain rate were investigated. According to Plougonven and Snyder^51^, internal wave increases are important for nutrient upwelling when the divergence divided by *f* is approximately 0.15–2.0; however, in the present case, the divergence was 1–2 orders of magnitude smaller than the value of approximately 0.15–2.0 (not shown). Ito et al.^52^ found that a lateral strain rate of approximately 1.6 × 10^–5^ s^−1^ is important for frontgenesis, whereas in the present case it was also one to two orders of magnitude lower (not shown). Thus, the results suggest that internal waves and submesoscale frontgenesis are unlikely to be driving significant nutrient upwelling in the offshore area in the present case. Therefore, the above discussion directs the scenario that coastal waters promote high-nutrient water and accelerate phytoplankton bloom.

From the above discussion, it was not possible to postulate the factors that prevented chlorophyll-*a* from reaching a maximum in 2014 in the maxima in low-salt transport. Moreover in 2014, strengths of the anticyclonic eddy and OBTWC (Fig. 4a,b) were also maximal, even though the PAR and *K*_*d*_ were also large (Fig. S2). It was not possible to estimate the factors that prevented chlorophyll-*a* from reaching a maximum (Fig. 4b) in 2014, other than the factors mentioned above; it is likely that in 2014 there were factors other than those mentioned above.

The reasons why chlorophyll-*a* was higher in 2011 are also not clear from the discussion of this study. Although the present study can only address processes of fluctuations in light and turbidity in the surface layer using satellites, there may be processes of these fluctuations in the subsurface layer. Further studies are expected to include CTD observations with light and turbidity sensors, as well as glider observations and water sampling observations.

## Concluding remarks

**Figure 6:**
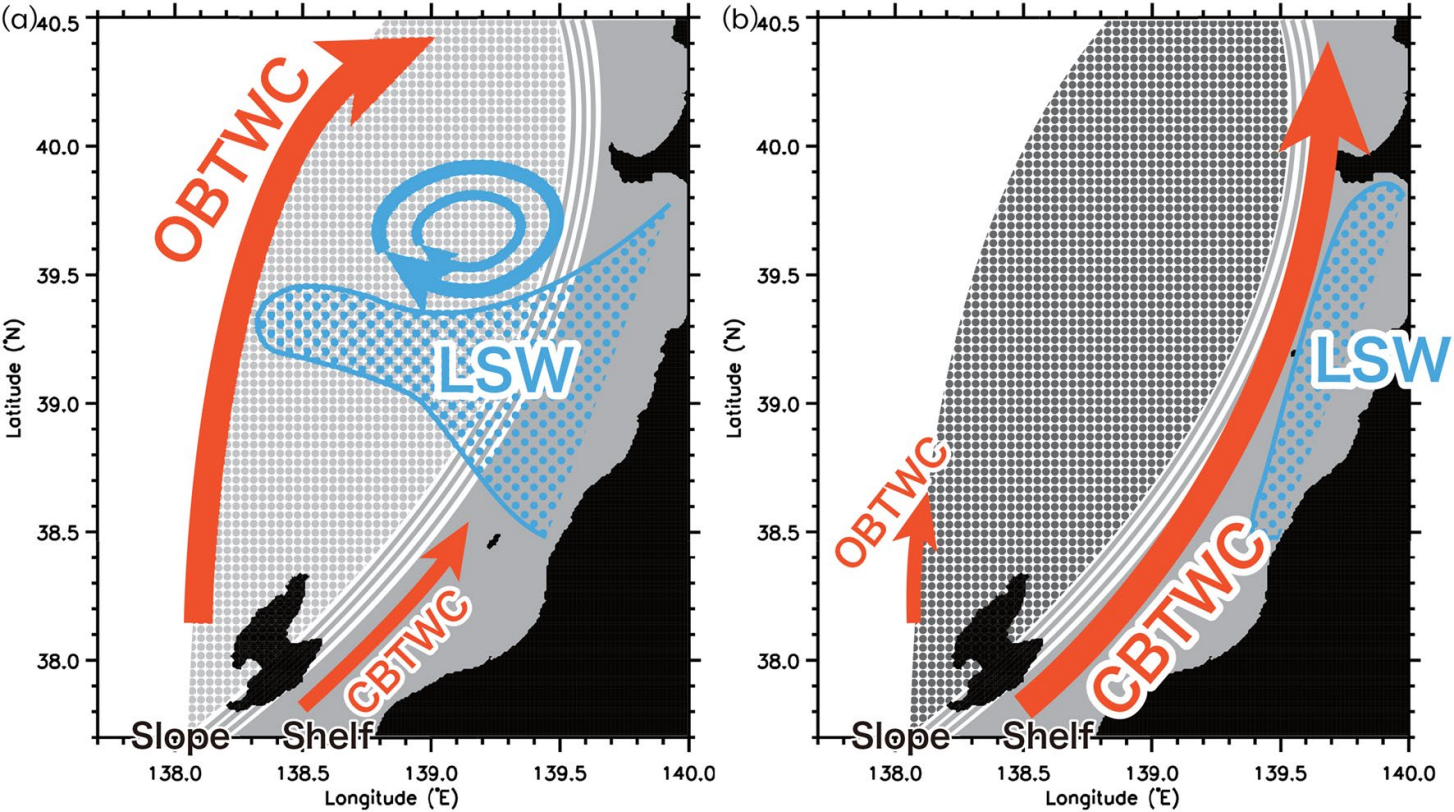
Schematic plot representing the relationship between the offshore spread of low-salinity water (LSW) from coastal areas and eddies, background offshore salinity field, and a coastal (an offshore) branch of the Tsushima Warm Current (CBTWC (OBTWC)). Dotted light (heavy)-gray region represents a background offshore relatively low (high)-salinity water.

Here, we illustrated the relationship among the offshore spreading of coastal low-salinity water, Tsushima Warm Currents, and mesoscale eddies (Fig. 6). The complementary analysis of the numerical experimental results and in-situ observation data showed that the low-salinity water in the offshore region observed by the glider in 2016 originated in coastal waters and was efficiently transported to the offshore region under the influence of coastal-to-offshore oceanic structures such as mesoscale eddies. Meanwhile, preconditions can potentially block the spread of coastal water such as the freshwater discharge from Changjiang River, the atmospherically forced cooling in the previous winter, and the precipitation over Japan.

We also found that low-salt transport was highly variable over time (2016 was a significant year). This is significant because coastal water has an entirely different quality than ocean water and provides buoyancy, which can affect the interannual and medium- to long-term variability of marine ecosystems over a wide area of the ocean through the promotion of elemental cycles and springtime phytoplankton blooms. The abundant nutrient content of coastal waters is rapidly consumed and depleted by phytoplankton, and phytoplankton blooms are generally restricted to the coastal zone. We identified a novel biological production mechanism whereby a strong northwestward flow effectively transports coastal water to the offshore region while maintaining relatively high nutrient levels in speculation, which can also enhance primary production in the distant offshore region. We believe that presenting a new biological productivity mechanism, including its applicability to other seas, will have a significant impact.

The ULSW observed in offshore areas, hundreds of kilometers from land-using gliders, was shown to originate from the Japanese coast. The spreading process of coastal waters associated with mesoscale eddies has also been elucidated by analyzing and comparing the processes of each year. The next step is to clarify the physical and dynamic mechanisms of cross-shore transport. Investigating this using an idealized numerical model from which the physical essence can be extracted would be helpful.

## Methods

### Ocean reanalysis dataset

We used a Japanese coastal ocean reanalysis dataset, “MOVE/MRI. COM-JPN Dataset”, for analysis^53^. The ocean model data assimilation system used to create this dataset was developed by the Meteorological Research Institute. It comprises a Japanese coastal model (MRI.COM-JPN^54^) and a four-dimensional variational data assimilation system. The OGCM used for MRI.COM-JPN is the Meteorological Research Institute Community Ocean Model Ver.4^55^. The model domain was the entire coastal SJ, spanning from 117° to 160° E and 20° to 52° N. The horizontal resolution was 1/33° in the zonal direction and 1/50° in the meridional direction, corresponding to a distance of approximately 2 km. The model has 60 levels in the vertical direction, with the thickness increasing from 2 m at the surface to 600 m at 6300-m depth. The physical schemes of the model, such as the high-precision advection scheme, were selected to be suitable for high-resolution modeling. In addition, a tidal scheme with eight main tidal constituents and an inverse barometer effect was incorporated for coastal modeling. The surface boundary conditions were provided by the hourly MSM dataset of the Japan Meteorological Agency for wind and sea level pressure and the 3-hourly JRA55-do dataset^56^ for radiation, precipitation, air temperature, and dew point temperature. The data contained in the JRA55-do dataset were used for the river runoff analysis. For the lateral boundaries of the model, a two-way online double-nesting method was used with a North-Pacific and a global model. Various satellite and in-situ observations were assimilated in the outer two models, and the assimilation increments obtained were used in the inner JPN model to constrain mesoscale variations. A four-dimensional variational method was adopted to assimilate short-term variations in the coastal seas. See Usui et al.^57^ and Hirose et al.^58^ for details on the data assimilation method and assimilated data.

Thus, a system based on the Japanese coastal model and four-dimensional variational data assimilation generated a dataset that could be combined with coastal observations. From the data distributed by the Meteorological Research Institute, we analyzed data from 2010 to 2017.

### Observational data

Ocean surface chlorophyll-*a *(mg m^−3^) were downloaded via FTP server of the GobColour Project. GlobColour data (http://globcolour.info) used in this study has been developed, validated, and distributed by ACRI-ST, France. Level 3 merged chlorophyll-*a* (Sea-Viewing Wide Field-of-View Sensor, Medium-spectral Resolution Imaging Spectrometer, Moderate-resolution imaging spectra-radiometer, and Visible infrared Imaging Radiometer), PAR, and Kd daily data with 1/24° grid was selected for this study and any grid with following conditions were not used; no measurement, invalid, land turbid, ice and cloud.

The current paths of the OBTWC were detected using the absolute dynamic topography (ADT) by applying the algorithm developed by Yabe et al.^13^. We used the monthly mean of the daily ADT and geostrophic velocity for cells at 0.25° × 0.25° in latitude and longitude, measured by satellite altimetry (e.g., TOPEX/POSEIDON, Jason 1-3, ERS-1/2, and ENVISAT). This dataset was distributed by the Copernicus Marine Environment Monitoring Service (https://marine.copernicus.eu).

Temperature (T) and salinity (S) data were collected almost every month by the R/V Senshu-Maru of the Akita Prefecture Fisheries and Marine Research Institute using a CTD profiler (red and black open circles in Fig. 1b,d, respectively). The measurements were collected on the tenth of each month. The CTD profiles were obtained primarily at 300 dbar with a vertical resolution of 1 dbar. The CTD (SBE 911plus during 2000–2012 and 9plus during 2013–2020; Sea-Bird Electronics, Inc.) observations were performed along a regular line.

We also used monthly precipitation and snowfall data from the Aomori, Akita, and Yamagata prefectures obtained from the Automated Meteorological Data Acquisition System (AMeDAS) operated by the Japan Meteorological Agency (https://www.jma.go.jp/jma/en/Activities/amedas/amedas.html). Blue circles in Fig. 1b depict the AMeDAS station.

The 10-m-height hourly wind velocity from the grid-point-value reanalysis data produced and distributed by Japan Meteorological Agency (http://database.rish.kyoto-u.ac.jp/arch/jmadata/data/gpv/netcdf/MSM-S) were used because it was necessary to analyze variations shorter than 1 day.

### Supplementary Information

Supplementary Figures.

## Data Availability

The field observation data were deposited in the Fishery Resource Conservation Data Repository of the Japan Fisheries Agency. These data will be available to the Japan Oceanographic Data Center (http://www.jodc.go.jp/jodcweb/JDOSS/index.html). Other datasets generated and/or analyzed in the current study are available from the corresponding author upon request.
