# Supplementary material for: Freshwater spreading far offshore the Japanese coast
Source: Sci Rep. 2024 Jun 24;14:14508. doi: 10.1038/s41598-024-63275-6 (PMC11196277; doi:10.1038/s41598-024-63275-6)
Supplement: Supplementary file 1 — Supplementary Figures. [file 41598_2024_63275_MOESM1_ESM.pdf]

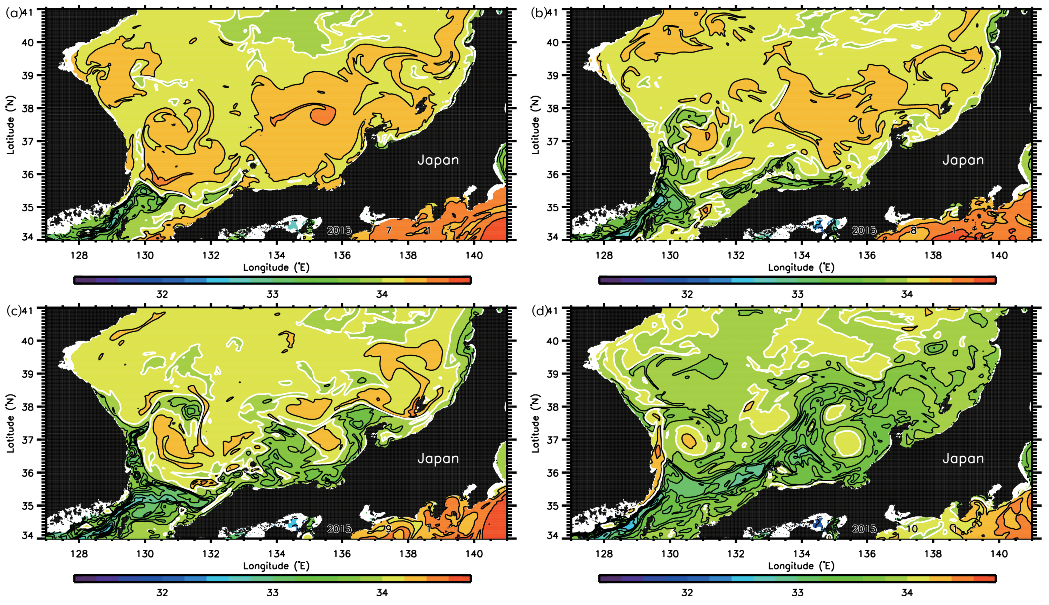

Fig. S 1: Model ULS at a depth of 40 m on 1 July (a), 1 August (b), 1 September (c), and 1 October (d) 2015 (thick white contours: salinity of 34.0). IDL 9.0 (https://www.nv5geospatialsoftware.com/Products/IDL) is used in generating the bathymetry based on the 1-km JTOPO30 data set provided by the Marine Information Research Center of the Japan Hydrographic Association (www.mirc.jha.or.jp/products/JTOPO30v2/).

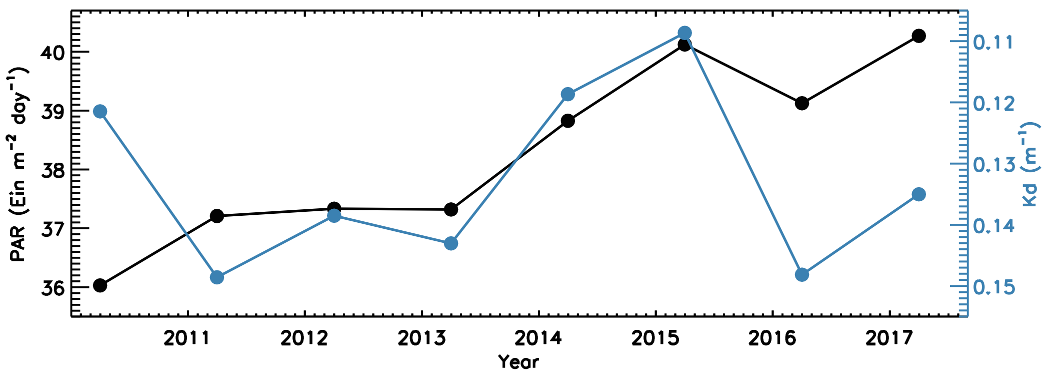

Fig. S 2: (a) Time series of interannual variations of the observed surface photosynthetically available radiation (PAR; black) and vertical diffuse attenuation coefficient at the wavelength of 490 nm (K_d; blue) averaged over the green rectangle of Figure 3a during April–May.

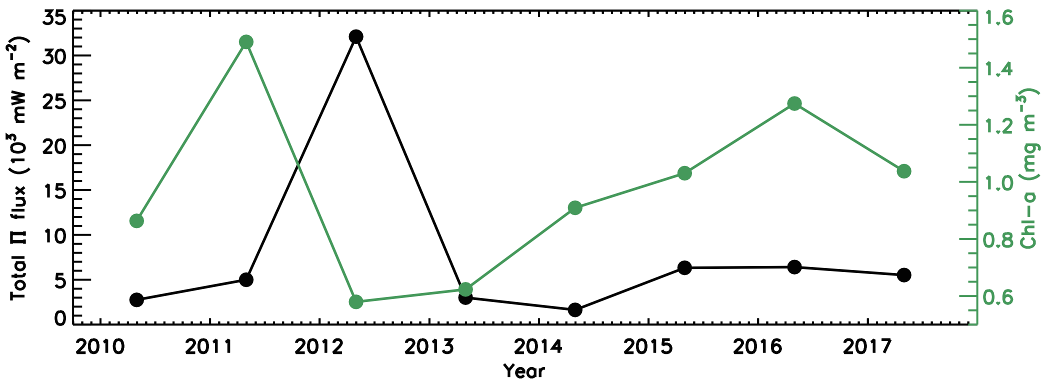

Fig. S 3: (a) Time series of interannual variations of the observed kinetic energy input Π of inertial oscillation into surface water at midpoint over the green rectangle of Figure 3a integrated during April–May (black) and sea-surface chlorophyll-a averaged over the green rectangle of Figure 3a during April–May (green).
